# A Network of Pathways Controlling Cellular Homeostasis Affects the Onset of Senescence in *Podospora anserina*

**DOI:** 10.3390/jof7040263

**Published:** 2021-03-31

**Authors:** Heinz D. Osiewacz, Lea Schürmanns

**Affiliations:** Institute for Molecular Biosciences, Faculty of Biosciences, Goethe University, Max-von-Laue-Str. 9, 60438 Frankfurt, Germany; schuermanns@bio.uni-frankfurt.de

**Keywords:** aging, autophagy, homeostasis, mitochondria, peroxisomes, *Podospora anserina*, quality control, signaling

## Abstract

Research on *Podospora anserina* unraveled a network of molecular pathways affecting biological aging. In particular, a number of pathways active in the control of mitochondria were identified on different levels. A long-known key process active during aging of *P. anserina* is the age-related reorganization of the mitochondrial DNA (mtDNA). Mechanisms involved in the stabilization of the mtDNA lead to lifespan extension. Another critical issue is to balance mitochondrial levels of reactive oxygen species (ROS). This is important because ROS are essential signaling molecules, but at increased levels cause molecular damage. At a higher level of the network, mechanisms are active in the repair of damaged compounds. However, if damage passes critical limits, the corresponding pathways are overwhelmed and impaired molecules as well as those present in excess are degraded by specific enzymes or via different forms of autophagy. Subsequently, degraded units need to be replaced by novel functional ones. The corresponding processes are dependent on the availability of intact genetic information. Although a number of different pathways involved in the control of cellular homeostasis were uncovered in the past, certainly many more exist. In addition, the signaling pathways involved in the control and coordination of the underlying pathways are only initially understood. In some cases, like the induction of autophagy, ROS are active. Additionally, sensing and signaling the energetic status of the organism plays a key role. The precise mechanisms involved are elusive and remain to be elucidated.

## 1. Introduction

*Podospora anserina* is a filamentous fungus that, in contrast to most other fungi, is characterized by a defined limited lifespan. Already in the 1950s it was reported that this ascomycete develops a well-defined senescence syndrome [1]. Depending on the strain, this syndrome occurs after a defined short period of growth (e.g., after 2–3 weeks): the pigmentation of the peripheral part of the thallus increases while the growth rate decreases until it comes to a complete stop and the thallus dies at the growth front. Subsequently, this phenotype was carefully investigated and it turned out to be under the control of environmental and genetic factors. Both nuclear as well as extranuclear genetic traits are active [2,3,4]. Later on, it was demonstrated that a genetic element located in mitochondria accumulates as a plasmid-like covalently closed circular DNA (plDNA). It behaves like a mobile element and gives rise to gross reorganization of the standard mitochondrial DNA (mtDNA). As a consequence, large parts of the mtDNA with a number of essential genes are deleted leading to deficiencies in mitochondrial biogenesis and function and death of the thallus at the hyphal tips [5,6,7,8,9].

Since this time, senescence in *P. anserina* was carefully analyzed and the fungus became a well-established model system in experimental aging research [10,11,12]. In particular, the analyses of a number of mutants, which live longer than the wild type, provided important clues and revealed insights into the mechanisms of lifespan control. This work unraveled a paramount role of mitochondria and of the cellular energy metabolism. One group of mutants (ex and mex) contained deletions of parts of the *PaCoxI* gene and, thus, an essential component of complex IV of the respiratory chain is ablated. In these mutants the expression of a nuclear gene coding for an alternative oxidase (PaAOX) is induced and respiratory deficiency is rescued [13,14]. As a consequence, the corresponding mutants are long-lived. The molecular basis of this example of mitochondrial-nuclear interactions, which requires signaling from impaired mitochondria to the nucleus and the activation of *PaAox*-specific transcription factors [15], became clear via the analysis of other mutants and uncovered an impact of ROS (for more details see below).

Subsequently, a number of different molecular pathways, involved in the control of cellular homeostasis, were identified which are effective in keeping the individual thallus functional over a longer period of time. However, when impairments accumulate beyond rescue limits, programmed cell death (PCD) [16] is induced and the thallus dies at the hyphal tips. PCD was found to be controlled by various factors like “apoptosis inducing factors” (AIFs) [17] and the activation of the two calcium-dependent metacaspases PaMCA1 and PaMCA2 [18,19,20]. During this process, the opening of a mitochondria transition pore (mPTP) plays a key role [21,22,23].

In this review, we focus on the network of interacting pathways and include recent studies that provide new perspectives to unravel the role of cellular homeostasis in aging and lifespan control of *P. anserina* in more detail. We put special emphasis on the bioenergetic role of mitochondria and include some ongoing work on the potential impact of mitochondrial ultrastructure regulation and on peroxisomes which, like mitochondria, are involved in the control of cellular energy metabolism. From these studies, it is clear that pathways controlling the quality and quantity of these organelles are crucial for cellular homeostasis. The involved pathways act at different cellular stages and need to be well coordinated by signal transduction pathways.

## 2. Generation, Balancing of Cellular Levels and Role of Reactive Oxygen Species

Previous research on mutants with a lifespan differing from that of the wild type was instrumental in the elaboration of mechanisms involved in the control of *P. anserina* aging and lifespan. Among the various long-lived mutants investigated, the grisea mutant, in which the nuclear encoded gene for the GRISEA transcription factor is not expressed due to a mutation in the single intron of this gene, provided a number of relevant data to understand the relevance of mitochondrial respiration. The grisea mutant is a copper-uptake mutant leading to deficiency in complex IV of the respiratory chain the function of which depends on the availability of copper as a cofactor. In this mutant, like in the mentioned ex mutants, the expression of *PaAox* is induced [24,25,26].

**Figure 1:**
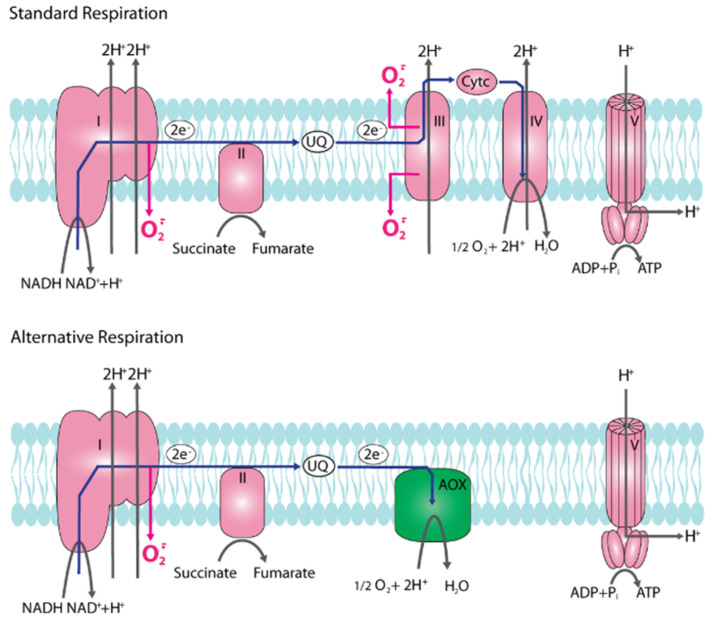
Scheme indication electron and proton flow in and through the inner mitochondrial membrane. Both, standard cytochrome c oxidase (IV) and alternative oxidase (AOX) dependent respiration are indicated. Standard respiration releases superoxide to both, the intermembrane space and the mitochondrial matrix. During alternative oxidation, superoxide is only released to the matrix at complex I. Since less protons are pumped to the intermembrane space via alternative respiration, the generated electron motive force is lower than at that of standard respiration and less ATP is generated at the F_o_F_1_-ATP-synthase (V).

**Figure 2:**
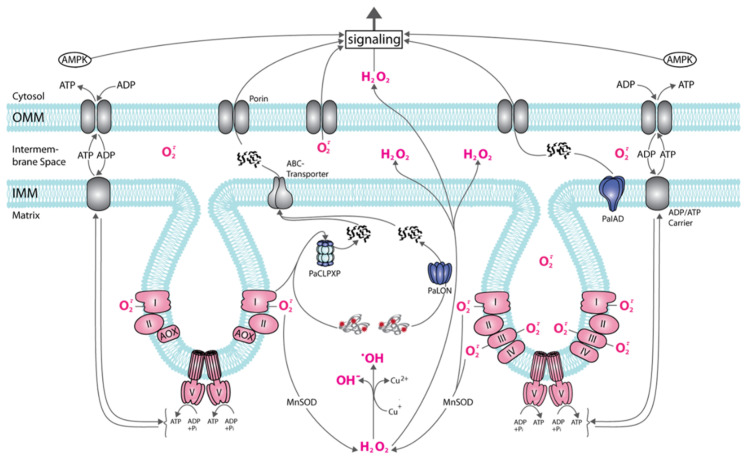
Age-related mitochondrial signaling in *P. anserina*. The scheme shows a part of a mitochondrion with two cristae formed by the invagination of the inner mitochondrial membrane (IMM). On the right, the standard respiratory chain is part of the IMM, on the left, the alternative respiratory chain with the alternative oxidase (AOX) is displayed. To note: at the standard respiratory chain superoxide is released into the inner space of cristae and into the matrix, at the alternative respiratory chain only into the matrix. In the matrix, superoxide (O^−^·) can be transformed into hydrogen peroxide (H_2_O_2_) that, in the presence of Cu (I) or Fe (II) (for simplicity only Cu (II) is shown) can form the highly toxic hydroxyl radical (·OH) or can pass the IMM and outer membrane (OMM) phospholipid layer reaching the cytosol. Superoxide in the intermembrane space can be released to the cytoplasm via porins. Damaged (red asterisks) or excessive proteins can be degraded by PaLON, PaCLPXP, PaIAP, or other proteases (not shown) to peptides that can be transported to the cytosol and are effective in the induction of responses. The energetic status (AMP/ATP ratio) of the cell can be sensed by AMPK and induces a variety of molecular responses.

The grisea mutant is long-lived and respires via an alternative respiration chain, by-passing complex III and IV of the respiratory chain (Figure 1). As a consequence, the formation of the superoxide anion (hereafter superoxide) is lower in the corresponding mutant. A disproportion reaction catalyzed by a superoxide dismutase (SOD) leads to hydrogen peroxide, a ROS that is able to pass the phospholipid bilayer of membranes and acts in the different cellular compartments. Hydrogen peroxide can be transformed to water via the activity of catalases, peroxidases or, in the presence of copper (I) or iron (II) (Cu^+^, Fe^2+^), can give rise to the formation of the hydroxyl radical for which no decomposition enzyme exists (Figure 2). This free radical is highly toxic and, together with the other ROS, causes damage to all kinds of cellular components like proteins, nucleic acids, and lipids. However, ROS (i.e., hydrogen peroxide) are also essential for cellular activities because, at low concentrations, they act as signaling molecules. Due to this dual function, it is essential to carefully balance cellular ROS levels which is achieved by controlling the generation of ROS and by scavenging them. Different components, enzymes, as well as nonenzymatic antioxidants (e.g., vitamins C and E, carotinoids, flavonoids, or polyamines), are involved in these processes [27,28,29,30,31,32,33,34,35].

As mentioned above, in heterotrophic eukaryotes a main site of ROS generation are mitochondria. The primary ROS is the superoxide free radical and mainly produced at complex I and III of the standard respiratory chain. At those complexes, superoxide molecules are released to the mitochondrial matrix, and at complex III to the intermembrane space as well (Figure 2). Due to its negative charge, superoxide is not able to cross biomembranes directly through the phospholipid bilayer. However, it can be released from the intermembrane space to the cytoplasm via anion channels (porins) in the outer mitochondrial membrane [36].

In one *P. anserina* study, it was demonstrated that respiration via the copper-independent alternative pathway results in a strongly reduced generation of superoxide, explaining the increased lifespan as a result of a reduction in ROS-induced molecular damaging [37]. This explanation can also be applied to other mutants which respire via the alternative oxidase [14,38]. As a consequence of by-passing complex III and IV of the respiratory chain, which are proton pumping membrane complexes, the electromotive force generated at the inner mitochondrial membrane is lower in mutants respiring via PaAOX than in strains using the standard PaCOX-dependent pathway and therefore less ATP is generated at the F_o_F_1_-ATP-synthase (complex V). The derived differences in the energetic status are responsible for the observed reduction in growth rate and impairments in the formation of female gametangia [14,25,38]. Although not analyzed in detail, sensing (i.e., the AMP/ATP ratio) and signaling of the energy status by “AMP-activated kinase” (AMPK), a central sensor and regulator of the cellular nutrient status, is involved in the underlying molecular pathways (Figure 2).

The effect of ROS scavenging on *P. anserina* was investigated in several studies. One series of studies analyzed the effect of the modulation of PaSOD3 levels, a manganese-dependent mitochondrial SOD (MnSOD). The abundance of this isoform was found to decline during aging [22]. Surprisingly, the overexpression of the corresponding gene did not result in lifespan extension but in a decreased lifespan and resistance against hydrogen peroxide [35]. In the mutant, it was found that several enzymes involved in cellular quality control were affected. These effects turned out to result from the increased generation of hydrogen peroxide by the increased abundance of PaSOD3 in the overexpressor [30,39].

In another series of studies PaMTH1, a protein encoded by a nuclear gene, was identified to accumulate during aging and turned out to be an *S*-adenosylmethionine-dependent methyltransferase [22,29,40]. During aging the protein is imported into mitochondria. PaMTH1 is able to methylate flavonoids with vicinal hydroxyl groups, which are prone to produce ROS in the presence of iron or copper [31,41]. The methylation of these groups via PaMTH1 thus prevents ROS generation by these compounds. In accordance with this function, overexpression of *PaMth1* leads to a protection of proteins against oxidation and an increase in lifespan [32,42].

Overall, the data demonstrate a damaging effect of ROS contributing to degeneration of *P. anserina* cultures. Processes leading to well-balanced levels of ROS are effective in the control of cellular homeostasis and act as pro-survival mechanisms.

## 3. Repair and Degradation of Cellular Components

### 3.1. Stabilization of mtDNA and Mitochondrial Base Excision Repair

Apart from pathways active in balancing the generation and scavenging of ROS preventing damage of cellular components, mechanisms evolved which repair damaged molecules or, if this is not possible, degrade them and resynthesize new and functional ones. The control of the integrity of DNA is well investigated in many biological systems in great detail. As mentioned, the mtDNA of *P. anserina* becomes greatly rearranged during aging. In this process, the first intron of the *PaCoxI* gene plays a key role which, after liberation and formation of plDNA, acts as a mutator via the induction of age-related mtDNA rearrangements [6,7,8,43]. In a first step, plDNA integrates either into a position directly downstream of the pl-intron in *PaCoxI* (“homing-like” transposition) or into other sites in the mtDNA (“ectopic” transposition). Thus, two intron copies are present in a single mtDNA molecule. Homologous recombination between these sequences may lead to the amplification of plDNA or of larger mtDNA circles. Those circles containing no origin of replication are subsequently lost. Significantly, although not formally proven, a protein with reverse transcriptase activity encoded by the pl-intron may be involved in transposition processes [44]. The described scenario is supported by various experimental data. For instance, in strains in which the pl-intron is deleted (e.g., ex and mex mutants) the mtDNA is stabilized [13,14]. Or, in the long-lived mutant AL2-1, the processes leading to the amplification of plDNA are delayed. This delay is linked to the presence of the linear plasmid pAL2-1, which encodes an RNA and DNA polymerase [45,46,47].

Apart from the processes counteracting gross mtDNA reorganizations, repair of subtle mutations is possible and likely to affect aging in *P. anserina*. In one study, a decrease of base excision repair (BER) activity was reported during aging of *P. anserina*. For one enzyme of BER, DNA glycosylase, activity was found to be higher in long-lived mutants with a lower ROS burden [48].

### 3.2. Degradation of Damaged Molecules and of Excess Components

During the lifespan of any organism, all kinds of cellular components change in quality and quantity. For instance, due to molecular stress (e.g., oxidative stress, heat stress) proteins may be oxidized or may aggregate and become impaired in function. Or, due to exogenous (e.g., nutrient deprivation) or endogenous conditions (e.g., developmental stages) their abundance needs to be adopted. This situation requires a dynamic system in which damaged or excess components can be degraded and, if necessary, be resynthesized again. In *P. anserina*, different pathways were shown to be active and have a significant impact on lifespan.

#### 3.2.1. Proteases

One series of studies were dealing with naturally occurring processes active in controlling cellular protein quality. These proteases degrade their substrate proteins to peptides which, via membrane protein complexes (e.g., ABC transporters, porins), are released from mitochondria to the cytoplasm and give rise to signaling (Figure 2) [49,50]. Here a great impact of mitochondrial proteases on lifespan control and senescence of *P. anserina* was uncovered. For instance, two complementary studies uncovered the role of the PaLON, the mitochondrial matrix LON protease of *P. anserina*. In a strain overexpressing *PaLon*, the abundance of carbonylated proteins (i.e., mitochondrial aconitase) was found to be decreased. No effects on vital functions like fertility or growth rate were observed demonstrating an increase of the healthy period of time, the healthspan, in the lifespan of the fungus [51]. In contrast, deletion of *PaLon* retarded growth and led to lifespan reduction [52]. Another mitochondrial protease is PaIAP, which is an ATP-dependent protease in the inner mitochondrial membrane. From studies in yeast and *Neurospora crassa* it is known that this protease is involved in the degradation of inner membrane proteins (e.g., cytochrome oxidase subunit 2, prohibitins 1 and 2 [53,54]). Deletion of *PaIap* resulted in an unexpected pronounced lifespan extension. More detailed analysis uncovered that this lifespan extension occurs when cultures are grown at standard laboratory temperature of 27 °C. At temperatures of 37 °C spore germination and fruiting body development were affected and lifespan was decreased [55]. It appears that PaIAP is part of a flexible system allowing survival under changing temperature conditions as they appear in nature.

Yet another mitochondrial protease is PaCLPP which forms a multiprotein complex with PaCLPX (Figure 2). The PaCLPXP complex consists of two hexameric rings of PaCLPP forming the proteolytic chamber in which proteins are cleaved to peptides. This part of the complex interacts with one or two hexameric rings of PaCLPX, acting as chaperones and introducing the proteins to be degraded into the proteolytic chamber. Thus, the complex, which is located in the mitochondrial matrix, structurally resembles the cytoplasmic proteasome.

The function of the eukaryotic CLPXP complex is currently only initially elaborated. In the nematode *Caenorhabditis elegans* the protease was demonstrated to be involved in the control of the mitochondrial unfolded protein response [56]. In *P. anserina*, evidence derived from a stringent substrate-trapping assay provided compelling evidence for a key role of PaCLPXP in controlling the mitochondrial energy metabolism [57]. Among the identified 19 high confident substrates, there were proteins of the pyruvate dehydrogenase complex, the Krebs cycle and the respiratory chain [57]. Some overlapping proteins were later also found in mammals and *Arabidopsis thaliana*. In particular, components of the N-module of complex I of the respiratory chain appear to be conserved substrates of CLPXP [58,59,60].

Surprisingly, in *P. anserina*, the deletion of the genes coding for *PaClpP* as well as *PaClpX* let to a pronounced extension of the lifespan. In addition, the double mutant is long-lived. In this mutant mitochondrial respiration is affected: oxygen consumption experiments revealed a general decline of respiration. In comparison to the wild type, the double deletion strain displays a significant increase in alternative respiration. Unexpectedly, ATP content was not changed in the double mutant [61]. The mutant phenotype of the *PaClpP* deletion strain was rescued to wild-type characteristics by the expression of the human *ClpP* gene identifying a conservation of the proteins from the two evolutionarily-distant species [61,62].

The 26S proteasome, although not located in mitochondria, was previously demonstrated to be active in the degradation of mitochondrial proteins via “mitochondria-associated degradation” (MAD) [63]. Moreover, research on mammalian cell culture or centenarians revealed a link between high proteasome activity and long lifespan [64,65]. An attempt to identify a potential role of the ubiquitin proteasome system (UPS) in aging of *P. anserina*, an age-related analysis of transcripts and proteins of specific components of the *P. anserina* proteasome were studied. No age-related differences in abundance were found. Moreover, a study using *Gfp-Cl1* transgene coding for the CL1 degron sequence fused to GFP, led to the interesting observation that after heat stress this potential substrate of the proteasome localized to the vacuole. In western blot experiments, the fusion protein was partly degraded leaving its GFP portion stably retained. Overall, this specific approach did not reveal evidence for the expected function of the UPS in quality control of the CL1 degron as a proteasomal substrate in *P. anserina* but instead suggested an efficient role of basal autophagy, the vacuolar degradation of proteins [66]. A role of autophagy in the control of aging was further suggested by a genome-wide transcriptome analysis of the *P. anserina* wild type. In this study, it was found that transcripts coding for the proteasomal subunits decreased in abundance at later stages in the lifespan while transcripts of the autophagic machinery increased [67] indicating that autophagy takes over quality control functions from the UPS in particular in older age. A later analysis basically verified this observation on the protein level but in addition identified that autophagy first increases in later age but finally decreases in very old age, in stages that were not investigated in the transcriptome analysis due to technical limitations [61,68].

#### 3.2.2. Vacuolar Degradation

First experimental evidence for an increase of macroautophagy, a form of autophagy in which cellular components are delivered via autophagosomes to the vacuole, was observed in a microscopic study investigating the formation of autophagosomes during aging of the *P. anserina* wild type in which a GFP-ATG8 fusion protein was expressed [69]. Under standard growth conditions on a minimal growth medium no or only few GFP-labeled autophagosomes were visible. In senescent cultures, a larger number of autophagosomes were observed. Moreover, under nitrogen-depleted conditions, under which autophagy is induced, autophagosomes were found to occur already in young cultures. These data verified a role of macroautophagy in the control of aging and the energetic status of *P. anserina*. Significantly, the ablation of PaATG1, a serine/threonine kinase that is essential for the formation of autophagosomes, led to a reduction of the wild-type specific lifespan and identified autophagy as a longevity-assurance pathway. The demonstration of the degradation of cytoplasmic superoxide dismutase 1 (SOD1) and the link to nitrogen starvation suggests that it is non-selective (bulk) autophagy, the degradation of cellular components in a portion of the cytoplasm delivered by autophagosomes to the vacuole, which is active to compensate age-related deficiencies in energy transduction and other impairments (e.g., quality control via UPS) as they appear in older age of *P. anserina*.

Another example for a compensatory, pro-survival function of autophagy was demonstrated in a *PaSod3* deletion mutant. Due to the ablation of the mitochondrial MnSOD (Figure 2), which is involved in mitochondrial superoxide scavenging, it was expected that the mutant is functionally impaired and short-lived. In fact, lifespan did not differ from that of the wild type. This unexpected phenotype was found to dependent on functional autophagy. In the *PaSod3* deletion strain, in contrast to the wild type, autophagy is induced already in young cultures. In addition, the induction of mitophagy, the selective vacuolar degradation of mitochondria, was strongly induced while non-selective autophagy in the mutant did not differ from that in the wild type [68]. Further on, in the same study, the effect of mild paraquat-induced external oxidative stress on the wild type and the *PaSod3* deletion strain revealed that addition to the growth medium of 20 µM paraquat, that gives rise to extra mitochondrial superoxide generation, had different effects on lifespan in the two strains. The wild type showed a strong increase in lifespan while in the mutant lifespan was decreased. These opposite effects are dependent on a functional autophagic machinery. Overall, these data can be explained by a hormetic (beneficial) effect of mild oxidative stress that is induced in the wild type. In contrast, in the mutant oxidative stress is already higher than in the wild type without the addition of paraquat due to the ablation of PaSOD3. In this situation, additional superoxide generation leads to excessive cellular oxidative stress which leads to autophagy-dependent cell death (ADCD) [16,68]. This kind of cell death is also observed in a short-lived mutant in which *PaCypD* coding for a peptidyl prolyl-cis, trans-isomerase (CYPD) a regulator of the mitochondrial permeability transition pore (mPTP) is overexpressed. Deletion of *PaCypD* leads to a decrease in autophagy in older age of the *P. anserina* wild type. Moreover, the study also demonstrated that PaCYPD is required for mitohormesis [23].

Taken together, autophagy appears to be a “double-edged sword”. Low stress results in hormetic, pro-survival effects of autophagy while excessive stress leads to death of *P. anserina* cultures via the induction of ADCD. These opposite outcomes can be triggered by exogenous factors. The polyphenol curcumin from *Curcuma longa* leads to a hormetic induction of autophagy and lifespan extension while the polyphenol gossypol from *Gossypium spec* leads to ADCD and a decreased lifespan [70,71].

Another example of the capacity to compensate impairments of other components of pathways in the control of cellular homeostasis was observed in the deletion mutants coding for the mitochondrial PaCLPXP complex (Figure 2). Counterintuitively, these mutants are long-lived with a constitutive induction of non-selective autophagy. Significantly, lifespan extension depends on a functional molecular autophagy machinery. Moreover, the relevance of autophagy was underlined by the observation that, in contrast to the wild type, autophagy was already induced in early life stages (four-day-old cultures) [61]. Overall, these data indicate intimate interactions of PaCLPXP and autophagy. A common function of non-selective autophagy and of CLPXP is the control of cellular metabolism. This link was further demonstrated in a recent study aimed to further characterize PaCLPXP. In this study the surprising observation was that the deletion of *PaSnf1* coding for the catalytic subunit of AMPK led to lifespan extension (Figure 2) [72]. The study revealed that PaSNF1 is required for autophagy, mitochondrial dynamics and respiration. Most surprisingly, the *PaSnf1/PaClpP* double deletion resulted in a synergistic effect with an even longer lifespan than that of the single mutants. The lifespan increasing effect was found to be stronger in strains of the mating type “minus” containing the *rmp1-1* allele of the *rmp1* gene that is closely linked to the mating-type locus. These data imply the interaction of completely different molecular pathways active in protein quality control, the sensing and control of cellular energy with the poorly characterized RMP1 protein, a protein that is involved in respiratory complex assembly and is likely active in mitochondrial translation [73,74]. The coordination of the corresponding pathways is unclear.

The impact of macroautophagy in *P. anserina* was further demonstrated in a mutant in which PaATG24 was ablated leading to a short-lived phenotype. In addition, growth rate and fertility are affected [75]. PaATG24 is a putative sorting nexin. Members of this evolutionary conserved protein family are involved in vesicle transport, membrane trafficking and protein sorting [76,77,78]. Deletion of *PaAtg24* leads to a changed morphology and size of vacuoles and a reduction of non-selective and selective autophagy. Mitophagy is reduced in the mutant and increases during aging. In contrast, general autophagy and pexophagy, the selective degradation of peroxisomes, is almost completely inhibited in the mutant and does not change during aging. Overall, the data uncovered membrane-regulated pathways involved in autophagy and lifespan regulation. The impact of pexophagy on aging and lifespan control is yet not analyzed but, since this organelle is also involved in energy metabolism and interacts with mitochondria, it is an interesting question whether or not and in how far these organelles are subject to age-related regulation and for biological aging.

## 4. Biogenesis and Dynamics of Mitochondria

**Figure 3:**
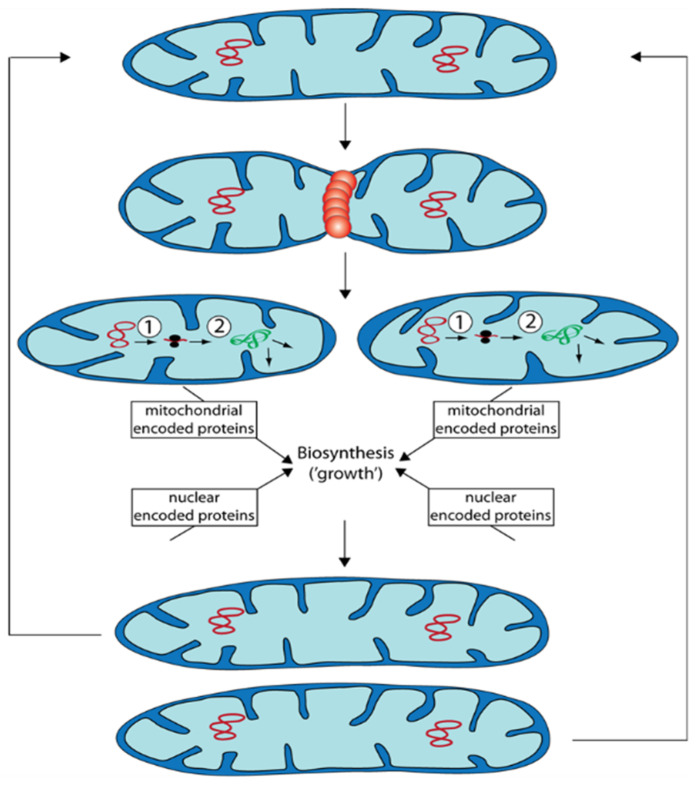
Propagation of mitochondria occurs via fission of prexisting organelles. After fission that is controlled by proteins (e.g., PaDNM1: orange restriction ring), the generated two mitochondria are smaller than the mitochondrion they are derived from. They “grow” depending on protein biosynthesis and on both, mtDNA (red circles) as well as nuclear DNA. Subsequently, they can divide again or they can fuse with other mitochondria. ①: Transcription of mtDNA. ②: Translation of RNA at mitochondrial ribosomes. Proteins (green) are subsequently sorted to the site of their function.

Mitochondria are semiautonomous organelles in which most of the approximately 1200–1600 proteins are encoded by nuclear DNA and only a few by mtDNA. Mitochondria are dynamic and change their morphology and ultrastructure depending on physiological constraints. Mitochondrial mass (size and number of mitochondria) changes during growth and development (Figure 3). This process is not the result of de novo synthesis of the organelle but by the biosynthesis of new components and their integration into existing mitochondria.

During “growth” of mitochondrial units, they form filamentous morphotypes that subsequently can divide into smaller units. These can fuse again to form filamentous organelles. Fission and fusion are genetically controlled by a number of proteins. Additionally, excess or functionally impaired (damaged) mitochondria can be removed by autophagy.

In addition to the processes of mitochondrial quality control discussed above the control of mitochondrial dynamics was demonstrated in *P. anserina* to have an effect on aging. Deletion of a gene coding for the dynamin-like protein PaDNM1, an essential protein involved in fission of mitochondria, led to an 11-fold increase in mean lifespan. Mitochondria of this strain had an strongly elongated morphology and even formed networks of fused filaments [79]. Only in very old cultures, mitochondria were found to be fragmented. Furthermore, in this strain no signs of typical reorganization of mtDNA found in the wild type occurred and the release of hydrogen peroxide was delayed to very old age. Lifespan extension was linked to an increase in resistance to the induction of programmed cell death. The relevance of *PaDnm1* for normal aging of the wild type is indicated by the increased transcription of the gene in old cultures [79]. Computational modeling integrating mitochondrial fission and fusion, ROS stress, and mitophagy revealed a positive impact of mitochondrial dynamics in situations when mitochondria are only marginally damaged. In contrast, deceleration of fission and fusion is an advantage to reach a long lifespan when damage of mitochondria passed critical limits [80,81].

## 5. Perspectives

As discussed above, a hierarchical network of pathways involved in the control of functional mitochondria and energy metabolism and impacts aging and lifespan of *P. anserina*. It is clear that other, yet not integrated, pathways and cellular compartments are involved in triggering degenerative processes in this species, which, at least in part, may be evolutionary conserved and, therefore, are also relevant in other species including humans.

### 5.1. Other Pathways Involved in Mitochondrial Homeostasis

Yet unexplored, but clearly relevant, are processes leading to mitochondrial biogenesis that are linked to a balanced expression of mitochondrial and nuclear encoded genes and the transport of proteins synthesized in the cytosol [49,82,83]. These proteins need to enter mitochondria via a sophisticated protein import machinery and delivered to the different mitochondrial sub-compartments where they have to be properly assembled to functional complexes (e.g., respiratory chain). The various steps of the underlying processes may be prone to age-related impairments and thus are certainly relevant for aging.

Other poorly investigated pathways are pathways that are involved in shaping mitochondrial ultrastructure. Age-related changes were demonstrated by electron cryotomography studies to occur during aging. It was found that the inner mitochondrial membrane changes from a tubular organization of cristae to the formation of vesicular units [84]. Moreover, it was shown that the inner membrane of vesicles at some sites come into contact with the outer membrane. At these sites, breakage of the outer membrane is thought to release mitochondrial vesicles. According to a model, the vesicles finally burst and release mitochondrial content, including mitochondrial copper that is stored in this organelle, into the cytoplasm. Subsequently, released excess copper may lead to the induction of the two metacaspases of *P. anserina* and finally to the induction of PCD [20]. The details of these processes are yet not formally demonstrated. However, in *P. anserina* the increase of cytoplasmic copper that may originate from mitochondria was demonstrated [85]. Interestingly, also during aging of human diploid fibroblasts an accumulation of cellular copper was reported [86], suggesting a conservation of molecular copper-related mechanisms.

Yet another factor involved in the typical cristae formation of juvenile mitochondria was demonstrated to be dimers of F_o_F_1_-ATP-synthase which are involved in curvature formation at the tip of the cristae (see above) [84,87]. It remains to be evaluated what finally leads to premature aging of *P. anserina* strains which are impaired in F_o_F_1_-ATP-synthase.

Another site of membrane curvature is the basic part of cristae, turned cristae junctions are controlled by the “mitochondrial contact site and cristae organization system” (MICOS). During aging and the reorganization of the inner mitochondrial membrane, these large protein complexes necessarily need to change. The impact of these protein complexes on aging has to be evaluated.

Additionally, lipid composition of mitochondrial membranes and the impact on mitochondrial function and aging is unexplored but an emerging field of interest. In particular, the lipid content of inner mitochondrial membrane, which evolved from the endocytosis of α-proteobacteria, differs from that of other typical eukaryotic membranes. In particular, the enhanced level of cardiolipin seems to be important. This lipid, a non-bilayer forming phospholipid of conical shape, has been implicated in the formation and stabilization of respiratory supercomplexes, which are more efficient in respiration than a chain of monomer respiratory complexes [88,89]. In this context the pathways involved in the formation of cardiolipin, which is located in the inner mitochondrial membrane, are of considerable interest to be unraveled.

### 5.2. Potential Role of Peroxisomes

**Figure 4:**
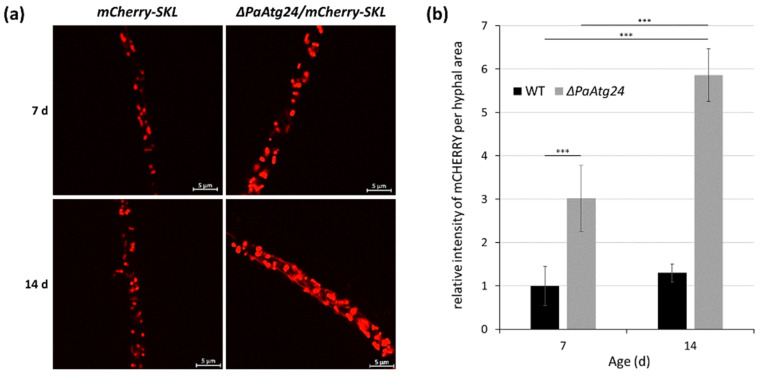
Age-dependent peroxisome abundance in the wild type and the *PaATG24* deletion mutant. (**a**) Fluorescence microscopic analysis of peroxisomes in *ΔPaAtg24* (*ΔPaAtg24/mCherry-SKL)* and control strain (*mCherry-SKL*) with peroxisomal reporter mCHERRY-SKL in seven- and 14-day-old cultures. Experimental conditions are described in [75]. (**b**) Quantification of relative intensity of mCHERRY signals per hyphal area. For this analysis 1491.1 μm^2^ hyphae of seven-day-old wild type, 1372.3 μm^2^ hyphae of 14-day-old wild type, 1433.4 μm^2^ hyphae of seven-day-old *PaAtg24* deletion mutant, as well as 1436.3 μm^2^ hyphae of 14-day-old *ΔPaAtg24* (*n* = 3, each) were analyzed. Error bars correspond to the standard deviation. For statistical analysis, the two-tailed *t*-test was used (*** = *p* ≤ 0.001).

In a previous study, investigating the role of PaATG24, it was found that deletion of *PaAtg24* reduces bulk autophagy, mitophagy and pexophagy [75]. Moreover, the number of peroxisomes, which slightly increase during wild-type aging, increases strongly in the short-lived *PaAtg24* deletion mutant suggesting a role of peroxisomes in the aging process in *P. anserina* (Figure 4).

A key function of peroxisomes is to degrade fatty acids via ß-oxidation leading to the formation of acetyl-CoA that finally is further metabolized (i.e., in the Krebs cycle in mitochondria). In most organisms, from yeast to humans, during the first step of this process acyl-CoA oxidase performed the oxidation of the fatty acid, which leads to trans-Δ^2^-enoyl-CoA and the formation of hydrogen peroxide as a byproduct. This molecule contributes to the cellular ROS load, to ROS scavenging, signaling, and via unbalanced conditions to molecular damaging.

There is considerable evidence that a disturbance in peroxisomal redox homeostasis affects mitochondrial function and redox balance. For instance, the inactivation of peroxisomal catalase (for more details see above) in human cells results in functionally impaired mitochondria, which lose their ability to maintain a membrane potential and synthesize ROS themselves [90]. On the other hand, it was shown that enhancing the activity of peroxisomal catalase has beneficial effects on mitochondria. It is described that during aging peroxisomal protein import of peroxisomal catalase is becoming particularly impaired [91]. Enhancing the effectivity of catalase import into the peroxisomes, reduces cellular hydrogen peroxide levels, as well as the number of senescent cells in a population, and reverses mitochondrial depolarization [92].

Interestingly, in some ascomycetous fungi, like *N. crassa* and *P. anserina*, no peroxisomal acyl-CoA oxidase was detected. Instead, the enzyme acyl-CoA dehydrogenase was found [93,94], which performs the same reaction as acyl-CoA oxidase but without the production of hydrogen peroxide. Without the production of this ROS, no detoxification system is needed and peroxisomes in *N. crassa* do not contain catalases [95]. In *P. anserina* the absence of all known catalases (PaCATA, PaCATB, PaCAT2, PaCATP1, and PaCATP2) has no effect on the usability of fatty acids and on the fungus’ lifespan [96]. These characteristics identify *P. anserina* as a great model to study peroxisomal processes beyond the different effects of ROS on the cell and unravel the peroxisomal ROS-independent role of peroxisomes on aging and development. For example, *P. anserina* is used as a model to study the Zellweger syndrome (also known as cerebro-hepato-renal syndrome). Patients of this hereditary disease cannot build functional peroxisomes. Malformations in the central nervous system, skeleton, liver and kidneys, and other organs, as well as a dramatically reduced lifespan characterize the syndrome [97]. Studies with a *P. anserina Pex5* deletion strain unraveled massive impairments of developmental processes leading to a strong reduction of the produced number of fruiting bodies. Interestingly, the morphology of mitochondria is also changed. While functional mitochondria of the wild type are described to be filamentous, the mutant shows round shaped and often aggregated mitochondria [98], which is a strong hint for interactions of mitochondria and peroxisomes. Such interactions are further supported by the fact that mitochondria and peroxisomes are both very dynamic organelles which partly use identical or homologous control elements. For instance, peroxisomal and mitochondrial fission both are controlled by the dynamin-related protein 1 (DNM1) [99,100]. In addition, for peroxisomes a DNM1-independent pathway mediated by the dynamin-related protein VPS1 is described [101]. However, in contrast to mitochondria, peroxisome biogenesis can occur also de novo from the endoplasmatic reticulum (ER) by sorting of peroxisomal membrane proteins to a specific region of the ER, from where pre-peroxisomal vesicles bud off. Afterwards peroxisomal matrix proteins are imported (for a current review see [102]).

Another similarity of mitochondria and peroxisomes is the use of isoforms of the LON protease that is responsible for degrading proteins damaged by oxidation. The activity of LON plays a critical role in maintaining function in both mitochondria and peroxisomes, because of its important role in removing oxidatively modified proteins and preventing their accumulation (reviewed in [103]). The role of peroxisomal LON in *P. anserina* is yet unclear but interesting, since *P. anserina* peroxisomes do not produce hydrogen peroxide during ß-oxidation and consequently oxidation of proteins may not be a critical issue in this species.

Since peroxisomes, in contrast to mitochondria, do not contain DNA or a gene expression machinery, all peroxisomal proteins are encoded by the nucleus. Peroxisomal proteins, which are synthesized in the cytoplasm, need to be properly transported to the site of their function in the peroxisomal membrane or the peroxisomal matrix. Thus, a protein import machinery is crucial for proper function of peroxisomes. In *P. anserina*, it has been shown that the absence of such proteins result in a variety of different impairments. For instance, the absence of PaPEX2 leads to a block of sexual development at the dikaryotic stage. Consequently, ascospore formation is blocked and the corresponding mutant strains are sterile [104]. In addition, it is known that peroxisomal dynamics are tightly regulated during the sexual development of the fungus. In the course of ascospore maturation, the number of peroxisomes decreases dramatically [105]. The observed elimination of peroxisomes is speculated to result from pexophagy. These data are linked to a recent study emphasizing a key role of pexophagy in the regulation of proper peroxisome numbers at different stages in the life of *P. anserina* [75]. The precise mechanisms involved in this regulation are yet not known but are a key focus of studies aimed to unravel the role of peroxisomes in aging and development.

## Data Availability

Original data from Figure 4 are available upon reasonable request from the corresponding author.
